# Disentangling biotic and abiotic drivers of intraspecific trait variation in woody plant seedlings at forest edges

**DOI:** 10.1002/ece3.7799

**Published:** 2021-06-21

**Authors:** Shilu Zheng, Bruce L. Webber, Raphael K. Didham, Chun Chen, Mingjian Yu

**Affiliations:** ^1^ School of Biological Sciences The University of Western Australia Crawley WA Australia; ^2^ Centre for Environment and Life Sciences CSIRO Health & Biosecurity Floreat WA Australia; ^3^ Western Australian Biodiversity Science Institute Perth WA Australia; ^4^ College of Life Sciences Zhejiang University Hangzhou China

**Keywords:** cage experiment, edge effect, habitat fragmentation, herbivory, intraspecific trait variation, plant architecture

## Abstract

In fragmented forests, edge effects can drive intraspecific variation in seedling performance that influences forest regeneration and plant composition. However, few studies have attempted to disentangle the relative biotic and abiotic drivers of intraspecific variation in seedling performance. In this study, we carried out a seedling transplant experiment with a factorial experimental design on three land‐bridge islands in the Thousand Island Lake, China, using four common native woody plant species. At different distances from the forest edge (2, 8, 32, 128 m), we transplanted four seedlings of each species into each of three cages: full‐cage, for herbivore exclusion; half‐cage, that allowed herbivore access but controlled for caging artifacts; and no‐cage control. In the 576 cages, we recorded branch architecture, leaf traits, and seedling survival for each seedling before and after the experimental treatment. Overall, after one full growing season, edge‐induced abiotic drivers and varied herbivory pressure led to intraspecific variation in seedling performance, including trade‐offs in seedling architecture and resource‐use strategies. However, responses varied across species with different life‐history strategies and depended on the driver in question, such that the abiotic and biotic effects were additive across species, rather than interactive. Edge‐induced abiotic variation modified seedling architecture of a shade‐tolerant species, leading to more vertical rather than lateral growth at edges. Meanwhile, increased herbivory pressure resulted in a shift toward lower dry matter investment in leaves of a light‐demanding species. Our results suggest that edge effects can drive rapid directional shifts in the performance and intraspecific traits of some woody plants from early ontogenetic stages, but most species in this study showed negligible phenotypic responses to edge effects. Moreover, species‐specific responses suggest the importance of interspecific differences modulating the degree of trait plasticity, implying the need to incorporate individual‐level responses when understanding the impact of forest fragmentation on plant communities.

## Graphical abstract

Edge effects can drive intraspecific variation in seedling performance in fragmented forests. Using a highly replicated seedling transplantation cage experiment at different distances from the forest edge, we disentangled the relative abiotic and biotic drivers of seedling intraspecific variation. We found that abiotic and biotic drivers have additive and species‐specific effects on rapid directional shifts in the performance and intraspecific traits of woody plants at forest edges from early ontogenetic stages.

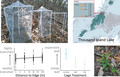

## 1 INTRODUCTION

Despite global recognition that we must conserve primary forests (Mackey et al., 2015), the rate of forest loss and fragmentation is still increasing (Newbold et al., 2015), such that degraded and regenerating forests are now the dominant vegetation types in most regions of the world (Hansen, 2013; Matos et al., 2020; Poorter et al., 2016; Zhu, 2002). In the regeneration pool at degraded forest edges, relative seedling performance plays a central role in forest succession, shaping future plant community composition, and ecosystem functioning (Lipoma et al., 2019). At the same time, the selection pressures acting on seedlings are themselves strongly influenced by the spatial context of forest fragmentation (Haddad et al., 2015; Matos et al., 2020). As one of the major abiotic factors limiting survival and growth of tree species in regenerating forests (Chazdon et al., 1996), light availability can be dramatically higher at forest edges compared to the interior (Didham & Lawton, 1999; Schmidt et al., 2017). Consequently, such altered spatial patterns of understory light can have profound effects on woody plant regeneration and performance (Chazdon et al., 1996), leading to altered functional composition of plant communities at forest fragment edges compared to the interior. For example, studies have tested whether altered environmental conditions at edges, including increasing light availability, drive “retrogressive succession” of forest edge communities toward an alternative stable state favoring species with early‐successional trait features (Ewers et al., 2017; Gascon et al., 2000; Laurance et al., 2006; Tabarelli et al., 2008). So far, the focus of studies on shifting functional composition of plant communities has been on interspecific turnover, from shade‐tolerant species in the forest interior to light‐demanding pioneers at the edge, rather than on intraspecific trait variation. However, performance, growth form, and functional traits of individual plants within the same species can also shift in response to altered abiotic and biotic conditions from the forest interior to the edge.

Changes in environmental conditions within fragmented forests, such as understory light availability, can not only influence seedling survival (Chazdon et al., 1996) but also alter trade‐offs in aboveground biomass allocation, such as vertical growth versus lateral branching (i.e., architectural arrangement; Ackerly & Donoghue, 1998; Küppers, 1989). For example, at the intraspecific level, seedlings growing under heavy shade in the forest interior may invest more heavily in lateral branching to avoid self‐shading (Valladares & Niinemets, 2008). Meanwhile, within species, leaf traits of seedlings can also shift along the leaf economic spectrum (Reich et al., 1999; Wright et al., 2004) in response to edge‐to‐interior light availability gradients, leading to the development of leaves with low dry mass investment per unit leaf area and low leaf chlorophyll content in the shaded forest interior (Mitchell & Bakker, 2014; Poorter, 2001). In contrast, plants growing at forest edges can develop thicker branches (Watt et al., 2005) and/or dense and small leaves in response to higher levels of disturbance, more light, and higher temperatures compared to plants in the forest interior (Finer & Jenkins, 2012; Raymundo et al., 2019).

Additionally, herbivory is another crucial factor influencing seedling survival and growth. High levels of herbivory at early ontogenetic stages of plants can often be associated with high seedling mortality (Cadenasso & Pickett, 2001). Seedlings under intensive herbivory pressure may also exhibit greater complexity of crown architecture (Archibald & Bond, 2003; Martínez & López‐Portillo, 2003), may reduce leaf palatability by having high dry mass investment per unit leaf area, or may develop leaves with low dry matter content to minimize biomass loss (Coley & Barone, 1996; Elger & Willby, 2003; Stowe et al., 2000; Webber & Woodrow, 2009).

In fragmented forests, dramatic shifts in both abiotic environmental conditions and herbivory pressure frequently co‐vary at forest edges (de Carvalho Guimarães et al., 2014) and potentially have non‐additive effects on plant performance at the seedling establishment stage (Boege & Marquis, 2005). Within the same species, plants growing at open forest edges with more light and higher levels of disturbance typically have relatively high dry mass investment in leaves (Mitchell & Bakker, 2014; Poorter, 2001), such that they may be less vulnerable to herbivores than plants growing in the forest interior (Poorter & Rozendaal, 2008). For example, in a herbivore exclusion cage experiment, Badano et al. (2015) found that the impact of herbivory on seedling mortality was greater in the forest interior than the edge. However, when the combined effects of both herbivory and abiotic conditions were considered, overall seedling mortality was actually higher at forest edges than in the forest interior. Thus, it is possible that both edge‐induced light availability gradients and herbivory pressure can jointly influence seedling performance in fragmented forests in a non‐additive manner. However, most conclusions regarding edge effects on seedling performance have only drawn on correlative evidence from vegetation surveys, with limited experimental evidence for shifts in relative seedling performance across edge‐to‐interior gradients (but see Badano et al., 2015; Benítez‐Malvido et al., 2018). Moreover, no studies to date have disentangled abiotic and herbivory effects on intraspecific variation in plant architectural development or resource‐use strategies at early ontogenetic stages using an experimental approach.

Here, we conduct a herbivore exclusion cage experiment across edge‐to‐interior gradients in forests regenerating following disturbance, with multiple replicate edges on three habitat islands for each of four species with varying life‐history strategies. With this experiment, we aim to disentangle herbivory and edge‐induced abiotic drivers of seedling trait shifts at forest edges and investigate whether herbivory and abiotic drivers will have interactive effects on seedlings. We predict that within the same species, seedlings growing at the forest edge will develop more “expensive” leaves with lower specific leaf area (Poorter & Rozendaal, 2008; Valladares & Niinemets, 2008) compared to the interior due to higher light availability at forest edges (Didham & Lawton, 1999). Meanwhile, herbivory‐driven trait shifts are likely to vary in direction and magnitude between different species, either through development of “expensive” tough leaves with higher leaf dry matter content or through development of “cheap” soft leaves with lower leaf dry matter content to minimize biomass loss to herbivores (Stowe et al., 2000). As herbivory pressure can alter plant capacity to respond to changed light conditions (Salgado‐Luarte & Gianoli, 2011), we expect that herbivory pressure and edge‐induced light gradients will interact to drive non‐additive effects on seedling performance.

## 2 METHODS

### 2.1 Study site

The study sites were located in the Thousand Island Lake (TIL, or Qiandao Lake, 29°22′–29°50′N, 118°34′–119°15′E) in eastern China, a reservoir formed in 1959 by the construction of a hydroelectric station on the Xin'an River. Prior to the formation of the lake, trees in the region, including on the islands that now exist, were clear‐felled. Therefore, forest regeneration across the area started at a similar time and from similar initial conditions (Hu et al., 2011). Since the 1980s, this area has been well protected by law from human disturbance, and currently 88.5% of the total island area is covered by forests (the remainder of the area is in the flood zone and on tiny islands which are too small to support trees). Forests here are subtropical regenerating forests composed of c. 62 woody plant species dominated by Masson pine (*Pinus massoniana* Lamb.; Pinaceae) in the canopy and broad‐leaved woody plants in the understory (Liu, Coomes, et al., 2018). Over the course of the 12‐month experimental period from October 2018, the TIL region experienced typical weather conditions, which is a subtropical monsoon climate with hot, humid summers and cold and relatively dry winters (Hu et al., 2011).

### 2.2 Experimental design

**FIGURE 1:**
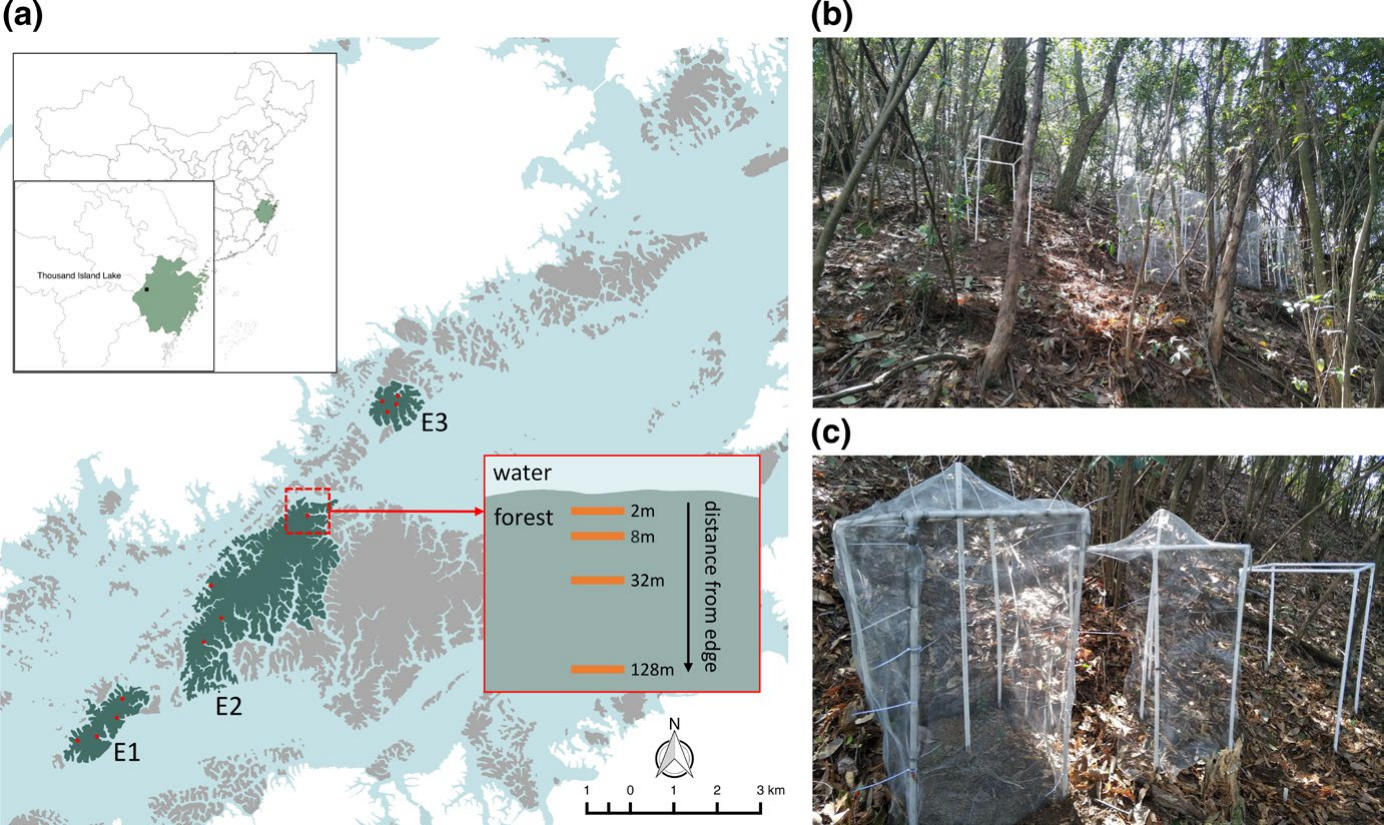
Experiment sites of this study. (a) In the main map of the study site, green areas represent sampling islands labeled with island codes (“E1,” “E2,” and “E3”), gray areas represent unsampled islands, white areas represent mainland, blue areas represent water, and red dots indicate experiment locations on each island. The inset panel shows the design of four experimental sites from forest edge to the interior at each experiment location. At each experimental site (b), each species was planted in three cages (c) spaced ca. 1–3 m apart: a “full‐cage” treatment (c; left cage), which is fully enclosed with stainless steel mesh; a “half‐cage” treatment (c; middle cage), which only has mesh cover on the cage top and on the side wall facing uphill against the slope; and a “control” cage without mesh (c; right cage)

In this study, three large islands (“E1”: 130.0 ha; “E2”: 750.0 ha; “E3”: 67.4 ha; Figure 1) were selected as experimental islands because their locations covered a large geographic area within the lake, they were subject to little human disturbance, and they were large enough to have long forest edge‐to‐interior gradients (i.e., with more than 128 m distance from the island edge to the interior). On the three selected islands, we used a stratified block design for the caging experiment. Considering potential aspect effects on seedling growth and leaf trait variation, experiment locations were set up on four aspects (i.e., topographic slopes facing the north, the south, the west, and the east) of each island. Within each aspect, four experimental sites were set up at distances of 2 m, 8 m, 32 m, and 128 m from the edge (on a log_2_ scale; i.e., 2^1^ m, 2^3^ m, 2^5^ m, and 2^7^ m; Ewers et al., 2011). At each of the four distances, three cages were set up c. 1–3 m apart in a loose spatial block: a “full‐cage” treatment, a “half‐cage” treatment, and a “control” cage (Figure 1) for each of four different plant species: *Schima superba* Gardn. et Champ. (Theaceae), *Vaccinium carlesii* Dunn (Ericaceae), *Rhododendron simsii* Planch. (Ericaceae), and *Quercus serrata* Thunb. var. *brevipetiolata* (A. DC.) Nakai (Fagaceae). This setup gave a total of 576 cages across all sites: 3 islands × 4 aspects × 4 distances from the edge × 4 species × 3 caging treatments. These four species were chosen because they had relatively high abundance in the island vegetation and were widely distributed across islands in the TIL region based on prior vegetation survey data. Also, these four species were qualitatively representative of the different plant life forms and different types of leaf life cycle in the wider plant community at the sites. Specifically, *V. carlesii* and *S. superba* are shade‐tolerant evergreen species, while *R. simsii* and *Q. serrata* are light‐demanding deciduous species; *V. carlesii* and *R. simsii* are shrubs, while *S. superba* and *Q. serrata* are trees.

In the full‐cage treatment, PVC piping cages (15 mm dia tubing) were fully enclosed with stainless steel mesh (0.28 mm wire thickness with 1.5 × 1.5 mm mesh holes) to protect seedlings from herbivory, with mesh walls being buried 15 cm in the ground to ensure herbivore exclusion. In the half‐cage treatment, each cage only had mesh cover on the cage top and one side wall facing uphill against the slope, to control for any potential shading effect of the mesh in the full‐cage treatment while allowing full herbivores access to the seedlings within the cage. The control cages were cages that only had the PVC cage frames without any mesh. The full‐cage treatment effectively reduced herbivore access to seedlings (median herbivory level in the full cages was 0.08%, compared to herbivory level 1.79% in control and half cages), and leaf herbivory in control and half cages was not significantly different (Figure S1). Illuminance (lux) within and outside each cage was measured concurrently with two light meters set up at 50 cm off the ground (i.e., approximately the height of seedlings), and the degree of illuminance reduction (c. 20%) caused by the cage was comparable between half‐ and full‐cage treatment (Figure S2). During the experimental period, edge sites had more open canopy and higher illuminance than experimental sites in the forest interior, especially during leaf flush in early spring (Figure S3).

During cage installation, all naturally occurring roots within each cage were removed to a depth of 20 cm, so that experimental seedlings would have minimal root competition with neighboring local plants over the duration of the experiment. Moreover, as evidence of pathogen damage was negligible at the sites, based on previous investigation and qualitative monitoring during the experiment (*pers. obs*.), we did not consider pathogen damage as a major driver of seedling performance. Thus, our experimental manipulation of planting locations and caging treatments has been designed to partition the relative influences of edge‐induced abiotic gradients and varying herbivory pressure on woody plant seedlings.

### 2.3 Seedling transplantation

Seedlings used in this experiment were germinated from seed and then raised in large pots in a nursery under a light shade cloth (c. 50% cover) before being acclimatized to the local site conditions for 3 weeks at the central research station on TIL. Herbivory was controlled manually prior to transplantation, by careful regular observation. At the time of transplantation into the cages, seedlings varied in age and height between species: *V. carlesii* were approximately 9 months old with a mean height of 8 cm (*SD* = 3 cm, range = 2–18 cm), seedlings of *R. simsii* were approximately 2 years old with a mean height of 17 cm (*SD* = 8 cm, range = 3–42 cm), seedlings of *Q. serrata* were approximately 2 years old with a mean height of 39 cm (*SD* = 17 cm, range = 9–70 cm), and *S. superba* were approximately 2 years old with a mean height of 51 cm (*SD* = 12 cm, range = 7–80 cm) at the time of transplantation. Although the species transplanted differed in their age and size, which is likely to influence growth rate and leaf trait values, we were not primarily interested in potential species‐level differences (or confounding factors that varied between species), but rather in the within‐species variance across treatments. In this regard, seedlings of the same species all came from a cohort that had similar age and size. Nevertheless, we randomized the allocation of individual plants into treatments to ensure that any genetic differences or intraspecific variation in seedling attributes (or initial degree of herbivory) at transplantation did not bias the outcome of the experiment (Figure S5, Table S1). In subsequent analyses, we also included measures of seedling early‐treatment size variation as covariates in all models (see Section 2.5). To reduce transpiration rates and potential seedling mortality in the event of dry weather, old senescing leaves were removed and large leaves were trimmed back by half before being transplanted to the field. All pre‐existing leaves on seedlings were marked discreetly on the under‐surface with a permanent marker pen before transplanting.

In late October 2018, seedlings were transplanted into the cages (Appendix S1) with three to four seedlings per cage. Because of the limited availability of *Q. serrata* seedlings in the nearby native plant nursery, it was only transplanted to experimental sites at 2 m and 128 m from the edge (i.e., a total of 72 cages). The other three species were transplanted to all preset cages (i.e., 144 cages per species). In total, 1,735 seedlings were transplanted into the field, including 537 *S. superba*; 528 *V. carlesii*; 441 *R. simsii*; and 229 *Q. serrata*.

### 2.4 Seedling inspection

In late February 2019 (i.e., late winter in the TIL region), 16 weeks after planting, seedlings were inspected to determine initial survival status, seedling size, branching architecture, and leaf traits (which we refer to here as “early‐treatment” seedling attributes) for later comparison with the final survey, or “post‐treatment” seedling attributes in October 2019.

For seedling size and branching architecture measurements, in the early‐treatment inspection, we measured seedling basal diameter at 1 cm above soil level with a vernier caliper (±0.01 mm), total height, and total branch length (from branch tip to branch attachment on the main stem) using a tape measure (±0.1 cm), and we counted the number of marked leaves (i.e., leaves that were present before seedlings were transplanted into the field cages) and live branches. In the post‐treatment inspections, seedling size was measured with the same protocol as in the early‐treatment inspection, except that the number of fully expanded new leaves that emerged in the field were counted (i.e., un‐marked leaves).

#### 2.4.1 Sampling for leaf traits

In our study, we focused on seedling leaf traits because they can be easily measured and their links with the resource‐use strategy of plants and with ecosystem functioning are already established (Reich et al., 1999; Wright et al., 2004). Five leaf functional traits were recorded for each seedling: Leaf Area (LA), Leaf Chlorophyll Content (LCC), Leaf Thickness (LT), Specific Leaf Area (SLA), and Leaf Dry Matter Content (LDMC), at both early‐treatment and post‐treatment stages.

The method of collecting each trait included the following steps. First, up to five marked leaves per seedling were randomly selected; then, each leaf was clamped carefully between two plastic boards in situ (one lower white board with scale markings and the other upper transparent board), and a leaf photograph was taken with a camera (Sony DSC‐W800/SC) positioned parallel to the surface of the board. Leaf photographs were further analyzed for leaf area as described below. During the early‐treatment inspection, for *R. simsii* seedlings that had large numbers of marked (i.e., pre‐existing to the trial) leaves, we randomly collected five whole leaves per seedling for functional trait measurements. For *V. carlesii* and *S. superba*, the number of leaves per seedling was low, so we only collected leaf disks (taken with a stainless steel leaf punch; c. 0.64 cm^2^ for *V. carlesii* and c. 0.90 cm^2^ for *S. superba*; <20% of leaf area per leaf avoiding the midrib) instead of whole leaves for functional trait measurements, in order to minimize any “sampling herbivory” impact on the seedlings. We note that taking whole leaves versus only sampling leaf disks might have differing impacts on subsequent trait development of seedlings, but we ensured that the sampling method was the same within each of the species and thus any differences in leaf sampling methods would not affect intraspecific patterns of trait variation in this study. For *Q. serrata* seedlings, the majority of leaves were removed before transplantation (because of large seedling size and poor root condition); therefore, only initial seedling size was recorded for *Q. serrata* (without leaf trait measurements). During the post‐treatment inspection, all fully expanded and toughened leaves that had grown after the transplant period (i.e., non‐marked leaves) for *V. carlesii*, *S. superba*, and *Q. serrata* seedlings were collected. For *R. simsii* seedlings, because of the large number of leaves on most seedlings (>50), we randomly selected up to three branches from each seedling, and all fully expanded and toughened leaves on each branch were used for functional trait measurements and herbivory quantification. For both early‐ and post‐inspection work, leaf samples were sealed within plastic bags and stored in an ice bag immediately after being removed from the plant and then brought back to the laboratory for leaf functional trait measurements within 6 hr.

#### 2.4.2 Leaf trait quantification

All sampled leaves were scanned for leaf area and herbivory quantification (for herbivory quantification methods see Appendix S2). Leaf scans as well as leaf photographs taken in the field (for *V. carlesii* and *S. superba* in the early‐treatment inspection) were processed with the Wanshen Leaf Processing System (version 2018; www.wseen.com), with leaf area measured as the total leaf area including apparently healthy lamina as well as damaged portions. If any leaf had missing leaf margins, the lost area was re‐established based on an estimation from the shape of other intact leaves from the same branch or the same individual. Leaf chlorophyll content was measured using a SPAD‐502Plus chlorophyll meter (Konica Minolta), as the average of Soil Plant Analysis Development (SPAD) values taken at three random locations on the (apparently) healthy lamina of each sampled leaf (or for *V. carlesii* and *S. superba* in the initial inspection, five random leaves in the field). Leaf thickness was measured with micrometer calipers (±0.001 mm) at three random spots on each leaf sample, avoiding leaf veins. For SLA and LDMC measures, each leaf sample was first weighed with an analytical balance (±0.001 g) to obtain leaf sample fresh mass, and then, it was dried at 80°C for 48 hr or more (until the difference between two consecutive measurements was less than 0.002 g) and weighed again for leaf sample dry mass. Then, the SLA of each leaf sample was calculated as the ratio of fresh area to dry mass, and LDMC was calculated as the ratio of dry mass to fresh mass.

### 2.5 Data analyses

#### 2.5.1 Seedling size and branching architecture responses

**FIGURE 2:**
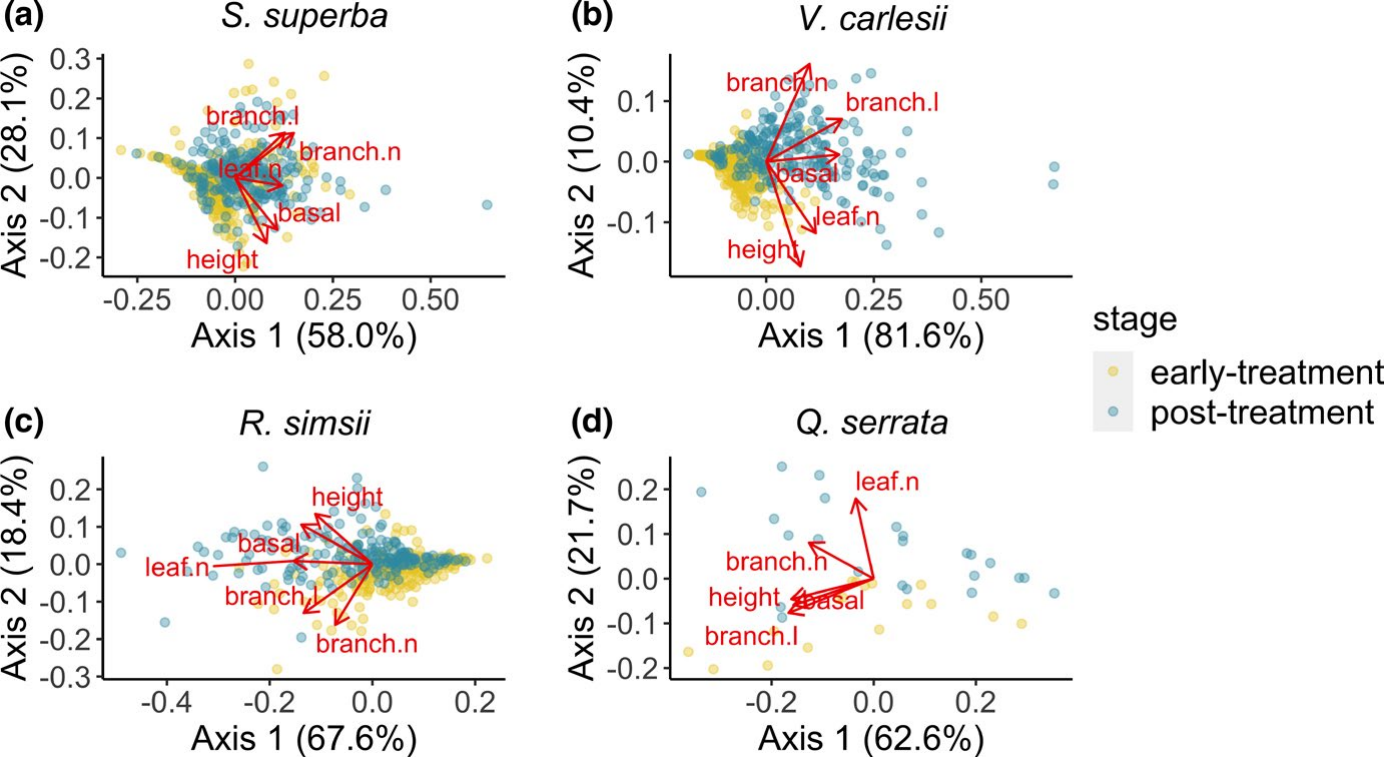
Ordination of multiple seedling size metrics (both at early‐ and post‐treatment stages) based on a separate principal coordinate analysis (PCoA) for (a) *Schima superba*, (b) *Vaccinium carlesii*, (c) *Rhododendron simsii*, and (d) *Quercus serrata*. Each dot represents a seedling measured at one time point, where yellow dots represent seedlings at the early‐treatment stage, surveyed in February 2019, and blue dots represent seedlings at the post‐treatment stage, surveyed in October 2019. Arrows represent principal component loadings for each size attribute; “height” is seedling height, “basal” is seedling basal diameter, “branch.n” is the number of alive first branches, “branch.l” is the total branch length, and “leaf.n” is the number of leaves

In order to identify the major sources of variation and trade‐offs among seedling size and branching architecture attributes within each of the four species, we carried out a preliminary principal coordinate analysis (PCoA) in the “stats” package (3.6.1; R Core Team, 2020) on seedlings at early‐treatment and post‐treatment stages, using a separate PCoA for each species. Note that each seedling was included twice in the PCoA analysis, as both early‐treatment seedling attributes and post‐treatment seedling attributes, so that changes in an individual's relative position within trait space through time (i.e., changes in PCoA axis values) would be directly comparable within the same trait hypervolume. The PCoA axis values for seedlings at the early‐treatment stage were treated as covariates in further size response analysis (see below), to account for the non‐independence between early‐ and post‐treatment PCoA axis values for the same individual. Prior to the PCoA analyses, all attributes were z‐transformed for each species. In the ordination results of seedling size and branching architecture, the first two axes explained c. 90% of the total size variation for each species (Figure 2). Specifically, for the four species, the five size attributes had loadings in the same direction (i.e., all positive or negative) on the first axis, and we refer to the first axis as “absolute size”; the second axis of each PCoA was associated with the trade‐off between seedling stem height and growth form complexity (e.g., branch number and branch length), and we refer to the second axis as “architectural complexity” (Figure 2).

For each species, we extracted the first two axes values in the PCoA as two major trade‐offs in seedling growth and applied linear mixed models (LMM) in the “nlme” package (Pinheiro et al., 2020) to test seedling growth response to experimental treatments. For each species separately, we treated the ordination axis values for seedlings at the post‐treatment stage in the PCoA results as the response variable, ordination axis values of seedlings at the early‐treatment stage as a covariate, and the interactions between cage treatment and distance to edge (log_2_ transformed) as fixed predictor effects. Random effects were specified in the models to account for the non‐independence of seedlings within the nested experimental design: multiple seedlings (i.e., seedling ID) within each cage, cages within each edge‐distance band, bands within each of the four aspects, and aspects within each island.

For each of the full models above, we developed candidate reduced models from all possible subsets of predictors, by dropping different fixed parameters. For subset model(s) that were well supported by the data (i.e., ∆AIC was <2 units greater than the top‐ranked model; Burnham & Anderson, 2002), we used the “model.avg” function in the “MuMIn” package (Bartoń, 2020) to obtain averaged coefficient estimates across these models.

#### 2.5.2 Seedling leaf trait responses

**FIGURE 3:**
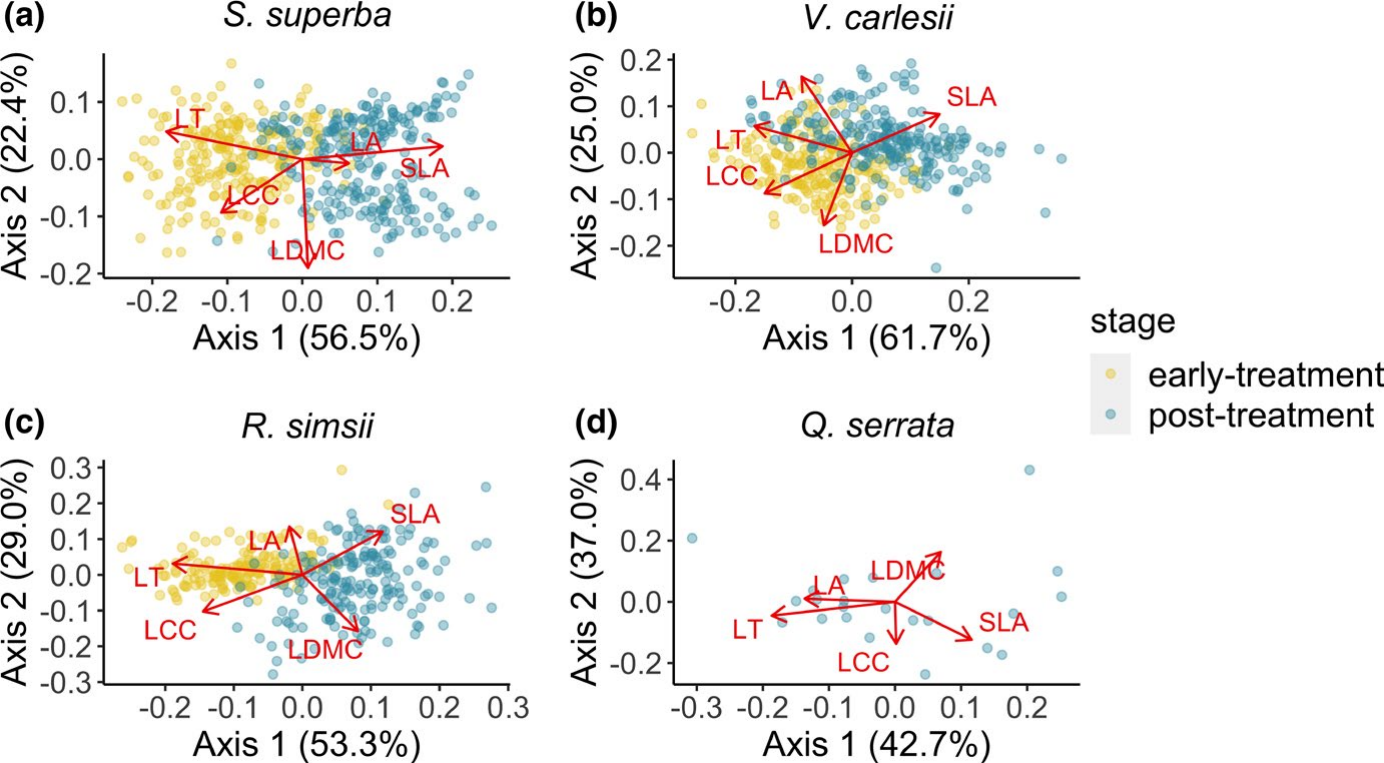
Ordination of multiple seedling leaf trait metrics (both at early‐ and post‐treatment stages) based on a separate principal coordinate analysis (PCoA) for (a) *Schima superba*, (b) *Vaccinium carlesii*, (c) *Rhododendron simsii*, and (d) *Quercus serrata*. Each dot represents a seedling measured at one time point, where yellow dots represent seedlings at the early‐treatment stage, surveyed in February 2019, and blue dots represent seedlings at the post‐treatment stage, surveyed in October 2019. Note that in (d), *Quercus serrata* seedlings at the early‐treatment stage did not have any leaves on stems, and the ordination is only based on post‐treatment trait values. Arrows represent principal component loadings for each size attribute; “LA” is leaf area, “LCC” is leaf chlorophyll content, “LT” is leaf thickness, “SLA” is specific leaf area, and “LDMC” is leaf dry matter content

Similar to seedling growth responses, we carried out a preliminary PCoA on seedling leaf traits of each species, at early‐treatment and post‐treatment stages, to identify the major sources of variation and trade‐offs among seedling leaf traits. Prior to the PCoA analyses, all leaf traits were z‐transformed for each species. In the ordination results, the first two axes explained c. 80% of total leaf trait variation for each species (Figure 3): The first axis in each PCoA was positively correlated with SLA and negatively correlated with LT and LCC, which is associated with the varying evolutionary strategies adopted by plants along a “fast”–“slow” spectrum of trade‐offs in biomass production of plants (Reich et al., 1999; Wright et al., 2004), and the second axis in each PCoA was correlated with LDMC, which is associated with leaf water content and the ability to tolerate stressful environments in plants (Pérez‐Harguindeguy et al., 2013; Figure 3). Thus, in this study, we refer to the first axis as a “resource‐use” axis and the second axis as a “stress tolerance” axis of functional trait variation.

For each species, we extracted the values of the first two axes of the PCoA as two major trade‐offs in seedling leaf traits and tested seedling trait response to experimental treatments with a LMM. Model structure for seedling trait responses to experimental treatments was similar to seedling size and architecture response models, except that for *Q. serrata* (which had no leaves on stems at the early‐treatment stage) the trait response models for this species did not have early‐treatment trait values as a covariate. Model selection was conducted with the same approach as for seedling size and architecture response models.

#### 2.5.3 Seedling survival responses

To test treatment effects on seedling survival for each species, we used generalized linear mixed models (GLMM) with a binomial distribution (logit link) in the “glmmTMB” package (Brooks et al., 2017) in R (3.6.1; R Core Team, 2020), testing the interaction between cage treatment and distance to edge (log_2_ transformed) on binary survival data (alive as “1” and not alive as “0”), with the nested experimental design reflected in the random effects components (see seedling size and architecture response model). Because of a very high (>90%) establishment mortality rate in *Q. serrata* seedlings, we applied a zero‐inflated model using the “glmmTMB” function (Brooks et al., 2017) for the *Q. serrata* seedling survival model.

Because initial seedling size at transplantation may influence seedling survival status in the field (Struve, 2009), we included initial seedling size as a covariate in GLMM models. As we had several correlated measures of seedling size (e.g., seedling height, basal diameter, and total branch length) that might have differential influences on seedling survival, we carried out a PCoA on early‐treatment seedling size attributes for each species and extracted the first two PCoA axes as covariates in the seedling survival model. For each species, the first two PCoA axes explained c. 90% of total seedling initial size variation (Figure S4). Model selection was conducted with the same approach as for seedling size and architecture response models.

## 3 RESULTS

### 3.1 Seedling size and branching architecture responses

**FIGURE 4:**
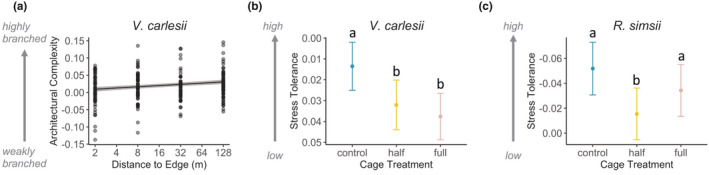
Seedling intraspecific trait variation in (a) architectural complexity (seedling size PCoA axis 2; Figure 2b) in response to distance to edge; (b) stress tolerance trait features (seedling trait PCoA axis 2; Figure 3b) in response to cage treatments for *Vaccinium carlesii* seedlings; and (c) stress tolerance trait features (seedling trait PCoA axis 2; Figure 3c) in response to cage treatments for *Rhododendron simsii* seedlings. Fitted lines are the predictions (±standard error) of model‐averaged coefficient estimates from linear mixed effects models with random effects reflecting the nested experimental design; treatments sharing a letter do not differ significantly (Tables S3 and S4)

After 8 months of growth in the field, all live seedlings had increased in absolute size and architectural complexity (i.e., more branching; Figure 2). In the LMM results, seedling architectural complexity (i.e., seedling size loadings on PCoA axis 2; Figure 2) of *V. carlesii* seedlings showed a significant response to distance to edge (Table S2). At the post‐treatment stage, *V. carlesii* seedlings growing in the interior showed more complex branching (i.e., larger ordination axis values) than seedlings growing at the forest edge (Figure 4a; Table S3). For *S. superba*, *R. simsii*, and *Q. serrata* seedlings, however, there were no significant differences in growth responses to edge effect treatments or cage treatments (Table S2).

### 3.2 Seedling leaf trait responses

Compared to leaf trait values at the early‐treatment stage, seedlings of *S. superba*, *V. carlesii,* and *R. simsii* developed new leaves with higher SLA and lower LT in the field at the post‐treatment stage (Figure 3). However, in our experiment, trait shifts along this “fast”–“slow” axis of biomass production trade‐offs (i.e., seedling trait PCoA axis 1; Figure 3) did not change significantly with edge effect treatments or cage treatments (Table S2). Meanwhile, trait shifts along the stress tolerance axis (i.e., seedling trait PCoA axis 2; Figure 3) varied significantly in response to cage treatments for the two shrub species, *V. carlesii* and *R. simsii*. Seedlings of *V. carlesii* in half‐ and full‐cage treatments invested less in leaf dry mass compared to seedlings in the uncaged control (Figure 4b; Table S3); seedlings of *R. simsii* in the half‐cage treatment invested less in leaf dry mass compared to seedlings growing in the other two cage treatments (Figure 4c; Table S4).

Meanwhile, for *S. superba* and *V. carlesii*, seedling trait change was associated with the change in seedling architectural complexity (seedling size PCoA axis 2), and seedlings developing faster‐growing leaves and investing less in leaf dry mass were associated with more complex seedling architecture (Table S7). As for seedling absolute size (seedling size PCoA axis 1), only *V. carlesii* showed associations between seedling size growth and changes in traits, where seedlings having leaves with resource‐acquisitive features showed slower growth in absolute size (Table S7).

### 3.3 Seedling survival responses

During the early‐treatment inspection in February 2019 (c. 3 months after transplantation), we recorded 93.0% survival overall, with 537 of 537 *S. superba*, 502 of 528 *V. carlesii*, 351 of 441 *R. simsii*, and 224 of 229 *Q. serrata* alive after the end of winter. By October 2019 after the warmer growth period, survival was much lower, with 243 *S. superba* (45.3%), 229 *V. carlesii* (45.6%), 191 *R. simsii* (54.4%), and 17 *Q. serrata* (7.3%) alive.

**FIGURE 5:**
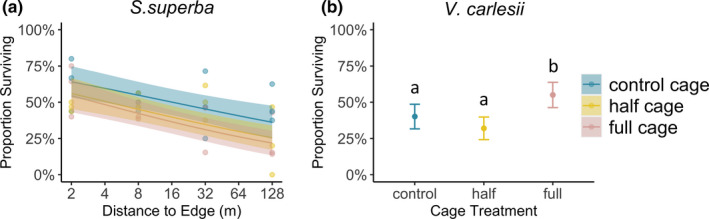
Seedling survival responses of (a) *Schima superba* from the forest edge to the interior, and (b) *Vaccinium carlesii* in different cage treatments. Fitted lines are the predictions (±standard error) of model‐averaged coefficient estimates from linear mixed effects models with random effects reflecting the nested experimental design; treatments sharing a letter do not differ significantly (Table S8)

In the generalized linear mixed effects models on seedling survival, only *S. superba* showed significant responses to distance to edge (Table S2). For *S. superba*, seedling survival decreased from island edge to the interior, irrespective of caging treatment (Figure 5a; Table S8). For *V. carlesii*, seedling survival showed a significant response to cage treatment (Table S2), with significantly higher survival in full cages where herbivores were excluded (Figure 5b; Table S8), while *R. simsii* seedling survival was not associated with cage treatment, and did not show significant responses to distance to edge in our study (Table S2). For *Q. serrata*, both distance to edge and cage treatment contributed to model fit (Table S2), but variance around model coefficients was high, giving low confidence in predicted effects (Table S8).

Within each experimental site, the associations between seedling survival and seedling trait change varied across species (Table S7). For *S. superba*, higher seedling survival was correlated with increasing investment in leaf dry mass (seedling trait PCoA axis 2), while for *R. simsii*, higher seedling survival within site was associated with less investment in leaf dry mass. For *V. carlesii*, seedling survival was negatively correlated with seedling trait shifting toward resource‐acquisitive features (i.e., high SLA and low LT; seedling trait PCoA axis 1).

## 4 DISCUSSION

Edge effects may alter the selection pressures acting on woody plant regeneration in forest remnants (Badano et al., 2015). Using a factorial experimental design of varying herbivory pressure at varying distances from the forest edge, we were able to disentangle the effects of edge‐driven changes in abiotic conditions versus variation in herbivory pressure on seedling performance. After one full growing season, both abiotic and biotic effects caused significant intraspecific shifts in seedling branching architecture, leaf economics traits, and survival. Surprisingly, however, the effects of different drivers were additive, rather than synergistic or antagonistic as might have been expected, and seedling responses varied strikingly across species. Here, we discuss the implications of these findings for future studies of the trait‐selection mechanisms operating in the regeneration layer of fragmented forest edges. Overall, the results of this study can help improve our understanding of the mechanisms underlying edge‐driven functional changes in woody plants and more broadly the influence of forest fragmentation on the functional structure of plant communities.

### 4.1 Abiotic drivers of seedling performance

Within one growing season, significant variation in seedling performance was found at different distances from the edge to the interior, irrespective of the herbivore exclusion effects. Edge‐driven variation in abiotic conditions led to a shift in seedling architectural trade‐offs for the shade‐tolerant shrub species, *V. carlesii*, with seedlings at the forest edge investing more heavily in vertical growth rather than lateral branching, compared to seedlings growing in the forest interior. Forest edges had more open canopy, and seedlings growing at forest edges therefore had greater accessibility to light than seedlings growing in the interior, especially during leaf flush in early spring (Figure S3). Accordingly, investment in lateral branching may help *V. carlesii* seedlings to intercept more light in the shaded forest interior (Valladares & Niinemets, 2008). *Vaccinium carlesii* is one of the most abundant species on islands in the TIL region (Liu et al., 2020), and its relatively high architectural plasticity in response to edge effects (compared with the other three species tested) could be one of the advantages that allows this species to thrive across a wide range of edge‐induced abiotic gradients in TIL (Cho et al., 2005). More generally, our results suggest that some woody plants can adjust their growth form along edge‐to‐interior stress and resource gradients, from a very young age. Such intraspecific variation in branching architecture may have important consequences via changes in the three‐dimensional geometry of forest structure, resulting in cascading effects on vertical stratification of microclimate (Didham & Ewers, 2014) and ecological processes such as competition between woody plant neighbors (Seidel et al., 2019), and animal foraging and nesting activities (e.g., Hinsley et al., 2009; North et al., 1999). Further studies are still needed to investigate the functional consequences of intraspecific tree architecture variation (Disney, 2019) and whether high plasticity in branching pattern can enhance individual survival and growth in fragmented forests.

In terms of leaf economics traits, by contrast, we did not observe significant variation in intraspecific responses to edge‐induced abiotic drivers, despite the fact that on islands in the TIL region we have already observed directional shifts in intraspecific leaf trait values of adult plants growing naturally at forest edge versus interior sites (Zheng, S., Didham, R. K., Yu, M., & Webber, B. L., unpublished data). It is possible that the observed intraspecific leaf trait shifts in adult plants between edges and interiors are caused by longer‐term local adaptation of populations in response to fragmentation (Quevedo et al., 2013). Moreover, because leaf trait variability may increase during leaf and plant ontogeny (Mitchell & Bakker, 2014; Niinemets, 2016), it is possible that individual trait responses to edge‐driven changes in abiotic conditions are slow to manifest throughout ontogenetic development in the species studied. A single sampling season may not be sufficient to detect recognizable changes in leaf resource‐use trade‐offs.

There was also no evidence that variation in seedling architectural or leaf trait responses translated directly into seedling survival variation among treatments. The only robust evidence for differences in seedling survival responses was for the evergreen shade‐tolerant tree species, *S. superba*, in which seedlings exhibited higher survival at forest edges compared to forest interiors. At face value, this result appears contrary to what might be expected for shade‐tolerant species, which might typically be expected to perform better in the forest interior. However, such a result is consistent with the prediction that seedlings of shade‐tolerant species often have better survival under intermediate light regimes, relative to high or low light conditions (Bloor & Grubb, 2003). In our study, experimental sites at the forest edges experienced higher light conditions relative to the forest interior (Figure S3). With moderate canopy cover, the light levels experienced by an individual seedling might not exceed thresholds that would compromise plant performance for *S. superba*. A previous study confirms such a situation, showing that seedlings of *S. superba* performed better under 15% of full light than under 5% of full light (Liu, Liu, et al., 2018). Coincidentally, the maximum canopy openness at edge sites in our experiment was c. 15%, potentially aligning with optimal light conditions for *S. superba* seedlings to persist, at least in the short term. It is worth noting that these results, based on a single growing season, may not hold over multiple seasons, particularly when combined with other factors such as the long‐term high level of wind disturbance and high temperature at the forest edge.

### 4.2 Herbivory effects on seedling performance

In our experiment, we found strong statistical evidence for herbivory effects on the two shrub species, *V. carlesii* and *R. simsii*. Seedlings of *R. simsii* showed significant variation in leaf trait response to herbivory exclusion treatments, irrespective of varying distances to forest edge, but showed no response in branch architecture or overall survival rates. Free from herbivory pressure, *R. simsii* seedlings growing in full‐covered cages developed smaller leaves with higher leaf dry matter investment, compared to seedlings growing under ambient herbivory pressure but similar abiotic conditions (i.e., in the half‐covered cage treatment). This result suggests that *R. simsii* seedlings under herbivory pressure have adopted the strategy of investing in cheap leaves with low leaf dry mass content to minimize the costs of herbivory and potentially enhance seedling fitness in the longer term (Stowe et al., 2000). Meanwhile, *R. simsii* seedlings growing in the uncovered control‐cage treatment developed leaves with significantly higher dry mass content than seedlings in the half‐covered cage treatment, which were exposed to similar herbivory pressure but different mesh cover conditions. Seedlings growing in uncovered cages have greater access to light and potentially higher wind disturbance due to cage artifacts, which can lead to higher leaf dry matter investment in plants (Dwyer et al., 2014; Valladares & Niinemets, 2008).

For the shade‐tolerant shrub *V. carlesii*, by contrast, seedlings showed significant variation in survival responses between herbivory exclusion treatments, irrespective of edge‐induced changes in abiotic conditions, but showed no response in either branch architecture or leaf trait variation. Compared to the other three species in our experiment, *V. carlesii* was the only species that showed a significant survival response to herbivory, having higher mortality in cages with herbivory access. Seedlings of *V. carlesii* were relatively younger (c. 9 months old) and smaller (c. 8 cm of above ground height) than seedlings of the other three species, and younger and smaller seedlings are known to be more vulnerable to a given amount of herbivory (Boege & Marquis, 2005; Chaneton et al., 2010).

### 4.3 No evidence for non‐additivity in the mechanisms driving edge effects

In this study, we did not find strong statistical support for an interaction between abiotic edge gradients and herbivory effects on seedling performance. One reason for this could simply be the low absolute levels of leaf herbivory observed on seedlings across all sites during the course of the experiment (Figure S1) and therefore the low power to detect an effect. Similarly, in the long‐term seedling experiment conducted by Benítez‐Malvido et al. (2018), the differences in the degree of leaf herbivory from the forest edge to the interior were subtle at the early stage of their experiment (c. 20 months), when seedlings were still young, with a limited number of leaves. In our experiment, within a single 8‐month growing season, we only observed c. 4% of seedling leaf function loss due to herbivory (Figure S1), which might be lower than any putative herbivory threshold that might induce leaf traits to change (Ito & Sakai, 2009). It is possible that after several growing seasons in the field, the accumulation of herbivory damage (Benítez‐Malvido et al., 2018) may lead to more evident herbivory‐driven changes in seedling performance and resource‐use trade‐offs along edge‐to‐interior gradients, which may then be reflected in altered leaf economics traits.

Although seedling survival of *V. carlesii* was compromised under herbivory pressure, trait responses of this species to herbivory were still negligible in this study. Instead, *V. carlesii* seedlings have preferentially allocated resources toward developing a more varied branching pattern in response to edge‐induced abiotic gradients (e.g., light availability). Such preferential investment in growth over defense suggests that *V. carlesii* may be more limited by resource acquisition and that maximizing photosynthetic capability, particularly via a branching structure more amenable to intercepting the scattered light in a forest understory, is a higher priority than investing more in defensive traits to reduce damage from herbivory (Boege & Marquis, 2005; Coley et al., 1985).

Taken together, we did not find evidence of significant interaction effects between herbivory and edge‐induced abiotic gradients on seedling responses. Further research on this area should prioritize long‐term field experiments on seedlings in fragmented forests (e.g., Benítez‐Malvido et al., 2018) in order to disentangle abiotic and biotic drivers of plant performance and trait variation at different ontogenetic stages.

### 4.4 Idiosyncratic responses across species

Across the four species we tested in our experiment, we did not observe a consistent general effect of either edge‐induced abiotic effects or varied herbivory pressure on seedling performance and intraspecific variation in architecture and leaf traits. Given that we deliberately chose species contrasting in their life history (deciduous vs. evergreen), habit (tree vs. shrub), and light regime preference (high vs. low light), this finding is perhaps not surprising. Previous studies have found that different species or even plants at different ontogenetic stages of the same individual plant can adopt different strategies even when under similar environmental stress or herbivory pressure (Boege & Marquis, 2005; Ochoa‐López et al., 2015; Webber & Woodrow, 2009). For example, Bloor and Grubb (2004) have found that seedlings of different shade‐tolerant species can have different trait plasticity under varied light conditions. Similarly, in the face of increasing herbivory rates, some plant species adopt defensive strategies such as spiny structures or tougher leaves to reduce herbivory (Coley & Barone, 1996), while other species adopt “tolerance” strategies, such as investing in “cheap” leaves with high turnover rate to minimize biomass loss to herbivores (Elger & Willby, 2003; Stowe et al., 2000). Species‐specific responses to edge‐induced abiotic gradients and varying herbivory pressure, such as those we observed in our experiments, highlight prominent interspecific differences in plant plasticity. Variable genetic and phenotypic plasticity among species can further influence species coexistence and community composition in an environment where forest fragmentation and degradation is an ever‐increasing concern (Turcotte & Levine, 2016).

## CONFLICT OF INTEREST

None declared.

## AUTHOR CONTRIBUTIONS

**Shilu Zheng:** Conceptualization (equal); data curation (lead); formal analysis (lead); funding acquisition (equal); investigation (lead); methodology (equal); project administration (lead); validation (equal); visualization (lead); writing–original draft (lead); writing–review and editing (equal). **Bruce L. Webber:** Conceptualization (equal); data curation (supporting); formal analysis (equal); funding acquisition (equal); methodology (equal); supervision (equal); validation (equal); visualization (equal); writing–original draft (equal); writing–review and editing (equal). **Raphael Didham:** Conceptualization (equal); data curation (supporting); formal analysis (equal); methodology (equal); supervision (equal); validation (equal); visualization (equal); writing–original draft (equal); writing–review and editing (equal). **Chun Chen:** Conceptualization (equal); data curation (supporting); formal analysis (supporting); investigation (equal); methodology (equal); validation (equal); visualization (equal); writing–original draft (equal); writing–review and editing (equal). **Mingjian Yu:** Conceptualization (equal); data curation (supporting); formal analysis (equal); funding acquisition (equal); methodology (equal); project administration (equal); resources (lead); supervision (equal); validation (equal); visualization (equal); writing–original draft (equal); writing–review and editing (equal).

## Supporting information

Supplementary Material

## Data Availability

Data are available from the Dryad Digital Repository: https://doi.org/10.5061/dryad.3ffbg79hz.
