# Supplementary material for: Disentangling biotic and abiotic drivers of intraspecific trait variation in woody plant seedlings at forest edges
Source: Ecol Evol. 2021 Jun 21;11(14):9728–40. doi: 10.1002/ece3.7799 (PMC8293732; doi:10.1002/ece3.7799)
Supplement: Supplementary file 1 — Supplementary Material [file ECE3-11-9728-s001.docx]

**Supplement**

**Appendix S1:** Seedling transplantation

Before transplanting the nursery raised seedlings into the island field sites, soil in each cage was prepared by removing roots and large rocks in the soil with a hoe, to enhance seedling survival rate in the field. To transplant each seedling, a hole was prepared in the soil ensuring that it was wider and about 40 mm deeper than the seedling pot. The nursery pot was then removed carefully, maintaining as much of the original soil around the roots as possible. Next, the seedling was placed into the hole, and the hole was filled with additional soil from the local cage site. The soil around the seedling was gently compacted, making a 2 cm high by 5 cm radius circular pit around the seedling for water conservation. Each seedling was watered gently with 500 ml of water as soon as it was transplanted to the field. After transplantation, each seedling of *V. carlesii*, *R. simsii* and *S. superba*, was watered with 500 ml of water twice in the first week, and then was watered once a week for the next three weeks to minimise transplant shock and improve establishment success (Struve, 2009). Considering the larger size and poorer initial condition of *Q. serrata* seedlings, they were watered twice a week for six weeks after transplanting. Note that watering was done for all seedlings within all treatments, only lasted for one month (prior to the end of December 2018) and ended before the seedlings growing season (i.e., the Spring season, commencing in late February 2019), and seedling inspections were conducted in February and October 2019 (i.e., ca. 3 months and 11 months after the final hand watering). Consequently, we do not expect to see any effect of watering on seedling treatment responses in our study. Natural litterfall on the top of cages was removed every three months in spring and summer and every month in autumn when leaves were falling from deciduous trees to ensure that cages were clear of obstructions and light was not impeded.

**Appendix S2:** herbivory quantification

During the final survey, we quantified herbivory for all sampled leaves. The functional consequences of herbivory for leaf photosynthetic function include not only leaf area lost through chewing herbivory, but also mining and skeletonizing. Therefore, relative differences in chlorophyll content in leaf tissue were used to estimate the loss of leaf photosynthetic function caused by herbivory. A SPAD-502Plus chlorophyll meter was used to indicate the relative amount of chlorophyll in the leaves by measuring transmitted red light (peak wavelength: ca. 650 nm, where the absorbance by chlorophyll is high and not affected by carotene, Lichtenthaler & Buschmann 2001) and infrared light (peak wavelength: ca. 940 nm, where the absorbance can be negligible) through a leaf, such that the resulting SPAD value corresponds to the amount of chlorophyll in the sample leaf. To quantify the loss of leaf function due to herbivory, SPAD values were recorded at multiple locations on each leaf that corresponded to different damage types, as well as at locations with (apparently) healthy leaf lamina on the same leaves. One SPAD value was taken for each damage type on the sample leaf (i.e., pathogen, mining, galling, and skeletonizing herbivory). If the damaged spot was smaller than the chlorophyll meter’s sensor window (i.e., 2 × 3 mm), the SPAD value for that damaged spot was estimated based on the average SPAD value for larger measurable spots of the same damage type on other leaves from the same branch or the same seedling individual. Total leaf area, and the partitioned relative areas of each damage type, were measured with the Wanshen Leaf Processing System. If any leaf had missing leaf margins, the lost area was estimated from the shape of other intact leaves from the same branch or the same individual. So, the percentage herbivory degree of each leaf (*H_leaf_*) was calculated as:

$H_{leaf}=1-\frac{{SPAD}_{green}\times{Area}_{green}+\sum_{i} {SPAD}_{i}\times{Area}_{i}}{{SPAD}_{green}\times\left( {Area}_{green}+{Area}_{loss}+\sum_{i} {Area}_{i} \right)}$;

where *i* indicates multiple damage types including pathogen, mining, and skeletonizing (note that galling herbivory was not observed on seedlings in this study). Then, the leaf herbivory degree for each seedling individual (*H_seedling_*) was calculated as the average of *H_leaf_* values from all sampled leaves of the seedling.

**Figure S1** Violin plots of actual leaf herbivory at the final stage in each cage treatment of all four species: *S. superba*, *V. carlesii*, *R. simsii*, and *Q. serrata*. In each box plot, the horizontal midpoint line indicates the median value, boxes represent the interquartile range, whiskers show the lowest and the highest values within 1.5 × interquartile range, and dots are outliers. Treatments sharing a letter do not differ significantly (Tukey’s HSD test, *P* < 0.05).

**Figure S2** Violin plots of relative illuminance for paired readings (lux) inside versus outside in each cage treatment. In each box plot, the horizontal midpoint line indicates the median value, boxes represent the interquartile range, whiskers show the lowest and the highest values within 1.5 × interquartile range, and dots are outliers. Treatments sharing a letter do not differ significantly (Tukey’s HSD test, *P* < 0.05).

**
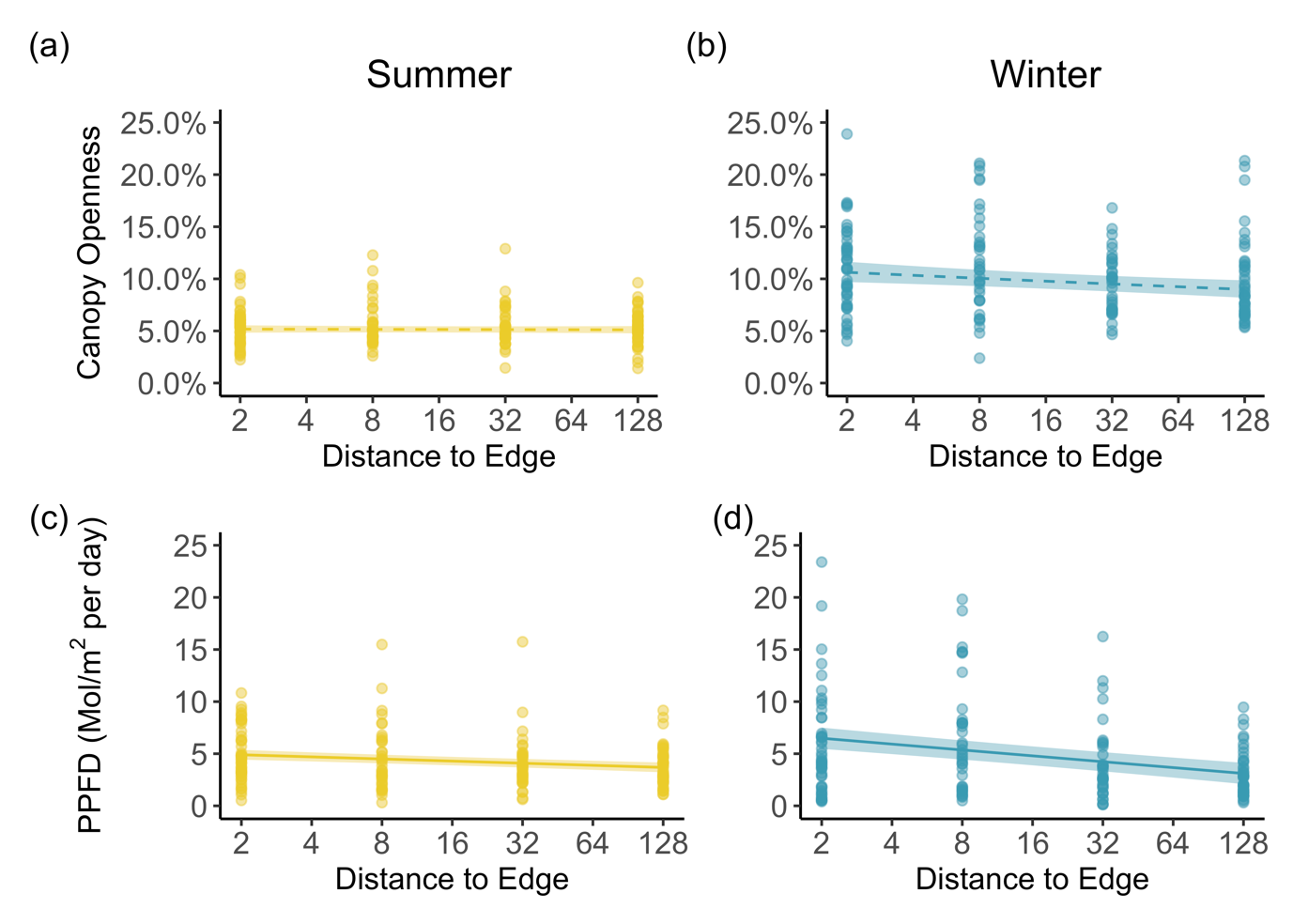
**

**Figure S3** Changes in (a, b) canopy openness and (c, d) photosynthetic photon flux density (PPFD) under the canopy in response to distance to edge during summer (a, c) and winter (b, d). For each block of three cages, two hemispherical photos were taken at the same locations, with one taken in late winter, February 2019 (when almost all deciduous trees had lost their leaves, and no new fresh leaves had yet emerged) and one taken in late summer, August 2019 (the peak of the growing season in TIL, with the highest degree of canopy closure), using a tripod-mounted Canon EOS 50D with a circular fisheye lens (Sigma 4.5 mm/F2.8) at 1.3 m height above ground. Canopy openness and PPFD under canopy during the growing season were calculated from hemispherical photos with WinSCANOPY^TM^ (Regent Instruments Inc.). Fitted lines are the model predictions (± standard error) from linear mixed effects models with nested experimental design as random effects (edge-distance bands within each of the four aspects within each island). Dashed lines (a, b) represent non-significant changes of canopy openness from the forest edge to the interior, while solid lines (c, d) represent a significant decrease in PPFD from the forest edge to the interior (*P* < 0.05).

**Figure S4** Ordination of seedling size at the early-treatment stage based on a separate Principal Coordinates Analysis (PCoA) for (a) *S. superba*, (b) *V. carlesii*, (c) *R. simsii*, and (d) *Q. serrata*. Each dot represents a seedling measured at the early-treatment stage. Arrows represent principal component loadings with size attribute: ‘height’ is seedling height, ‘basal’ is seedling basal diameter, ‘branch.n’ is the number of alive branches, ‘branch.l’ is the total length of all alive branches, and ‘leaf.n’ is the number of leaves.

**Figure S5** Seedling initial size distributions across sites for the four species. Axis.1 (a, c, e, g) and Axis.2 (b, d, f, h) are extracted from a PCoA of seedling initial size (see Figure S4). Although differing in initial size, seedlings were randomized by treatments across bands (from Band 1 at ca. 2 m from edge to Band 4 at ca. 128 m from edge) within each aspect (e, east; n, north; s, south; w, west) on each experiment island (E1, E2, E3). In each box plot, the horizontal midpoint line indicates the median value, boxes represent the interquartile range, whiskers show the lowest and the highest values within 1.5 × interquartile range, and dots are outliers.

**Table S1** Log-response ratio effect sizes (with 95% confidence interval in square brackets) for size and traits of seedlings at early-treatment stage. Here, ‘siz.1’ and ‘siz.2’ represent seedling size at the early-treatment stage loadings on Axis 1 and Axis 2 of the seedling size PCoA (Figure 3), ‘tra.1’ and ‘tra.2’ represent seedling traits at the early-treatment stage loadings on Axis 1 and Axis 2 of the seedling trait PCoA (Figure 4); ‘dist’ represents log_2_ distance to edge, ‘cage’ represents cage treatment; ‘A → B’ indicates that the effect size was calculated as the ratio of the mean value of treatment A compared with that of treatment B.

|  |  |  | **siz.1** | **siz.2** | **tra.1** | **tra.2** |
| --- | --- | --- | --- | --- | --- | --- |
| *S. superba* | cage | half → control | -0.40 [-4.68, 3.88] | 0.09 [-3.65, 3.83] | 0.12 [-3.26, 3.51] | 0.20 [-2.60, 3.00] |
|  |  | full → control | 0.16 [-4.51, 4.82] | 0.12 [-3.54, 3.78] | 0.19 [-3.16, 3.55] | 0.28 [-2.76, 3.32] |
|  | dist | 8m → 2m | -0.04 [-4.10, 4.03] | 0.79 [-2.92, 4.51] | 0.17 [-2.86, 3.19] | 0.37 [-2.67, 3.40] |
|  |  | 32m → 2m | 0.12 [-3.97, 4.22] | 0.16 [-2.66, 2.99] | 0.16 [-2.75, 3.07] | 0.04 [-2.42, 2.50] |
|  |  | 128m → 2m | 0.02 [-3.69, 3.74] | 0.35 [-2.34, 3.04] | 0.41 [-2.45, 3.28] | 1.17 [-0.84, 3.18] |
|  |  |  |  |  |  |  |
| *V. carlesii* | cage | half → control | 0.36 [-1.93, 2.64] | 0.15 [-1.27, 1.57] | 0.25 [-3.26, 3.76] | 0.26 [-2.41, 2.92] |
|  |  | full → control | -0.02 [-2.40, 2.35] | -0.25 [-1.78, 1.28] | -0.15 [-3.71, 3.41] | -0.11 [-2.89, 2.67] |
|  | dist | 8m → 2m | -0.10 [-2.17, 1.97] | -0.01 [-1.26, 1.24] | 0.02 [-3.10, 3.14] | -0.04 [-2.18, 2.10] |
|  |  | 32m → 2m | -0.01 [-2.04, 2.02] | 0.09 [-1.13, 1.30] | 0.09 [-3.02, 3.21] | 0.13 [-2.17, 2.43] |
|  |  | 128m → 2m | -0.07 [-2.43, 2.29] | -0.10 [-1.47, 1.27] | -0.20 [-3.15, 2.75] | 0.15 [-2.44, 2.74] |
|  |  |  |  |  |  |  |
| *R. simsii* | cage | half → control | -0.12 [-1.48, 1.24] | -0.12 [-2.31, 2.07] | -0.25 [-3.05, 2.54] | -0.24 [-1.44, 0.96] |
|  |  | full → control | 0.47 [-0.59, 1.54] | -0.19 [-2.51, 2.13] | -0.33 [-3.18, 2.52] | 0.33 [-0.74, 1.41] |
|  | dist | 8m → 2m | 0.62 [-0.37, 1.60] | -0.27 [-2.17, 1.64] | -0.32 [-2.72, 2.08] | 0.52 [-1.11, 2.15] |
|  |  | 32m → 2m | -0.31 [-2.60, 1.98] | -0.12 [-2.33, 2.09] | -0.47 [-2.78, 1.84] | -0.59 [-2.41, 1.24] |
|  |  | 128m → 2m | 0.49 [-0.63, 1.62] | 0.36 [-1.43, 2.15] | 0.26 [-2.12, 2.63] | 0.70 [-0.92, 2.33] |
|  |  |  |  |  |  |  |
| *Q. serrata* | cage | half → control | 0.91 [-1.00, 2.83] | 0.88 [-0.11, 1.86] |  |  |
|  |  | full → control | -0.48 [-2.79, 1.83] | -0.72 [-1.75, 0.30] |  |  |
|  | dist | 128m → 2m | 0.15 [-2.52, 2.83] | 0.56 [-0.54, 1.66] |  |  |

**Table S2** Results of model selection with maximum likelihood method (ML). Only candidate models with ∆AIC within 2 units of the top-ranked model are listed. Here, ‘surv’ represents survival, ‘siz.1’ and ‘siz.2’ represent seedling size at the post-treatment stage loadings on Axis 1 and Axis 2 of the seedling size PCoA (Figure 3), ‘tra.1’ and ‘tra.2’ represent seedling traits at the post-treatment stage loadings on Axis 1 and Axis 2 of the seedling trait PCoA (Figure 3); ‘dist’ represents log_2_ distance to edge, ‘cage’ represents cage treatment. ‘Model’ shows fixed predictor effects in each candidate models, ‘df’ represents degree of freedom, ‘logLik’ represents log likelihood, ‘BIC’ represents Bayesian information criterion, ‘AIC’ represents Akaike information criterion, ‘∆AIC’ is the difference in AIC values between the candidate model and the top-ranked model, ‘weight’ represents Akaike weight of the model among all possible subset models from the full model. Note that covariates of early-treatment size (for survival and size models) or traits (for trait models) are not shown in this table because they are always included in the models, except in the case of the trait models for *Q. serrata* that do not have early-treatment trait values available to use as covariates.

|  |  | Model | df | logLik | BIC | AIC | ∆AIC | weight |
| --- | --- | --- | --- | --- | --- | --- | --- | --- |
| *S. superba* | surv | ~ cage + dist | 10 | -314.023 | 690.9 | 648.0 | 0.0 | 0.458 |
|  |  | ~ dist | 8 | -316.753 | 683.8 | 649.5 | 1.5 | 0.220 |
|  |  | ~ cage * dist | 12 | -312.842 | 701.1 | 649.7 | 1.6 | 0.202 |
|  |  |  |  |  |  |  |  |  |
|  | siz.1 | ~ 1 | 7 | 275.764 | -513.2 | -537.5 | 0.0 | 0.545 |
|  |  |  |  |  |  |  |  |  |
|  | siz.2 | ~ 1 | 7 | 411.652 | -784.9 | -809.3 | 0.0 | 0.488 |
|  |  | ~ dist | 8 | 412.184 | -780.5 | -808.4 | 0.9 | 0.305 |
|  |  |  |  |  |  |  |  |  |
|  | tra.1 | ~ cage + dist | 10 | 331.484 | -608.3 | -643.0 | 0.0 | 0.298 |
|  |  | ~ cage | 9 | 330.391 | -611.6 | -642.8 | 0.2 | 0.271 |
|  |  | ~ 1 | 7 | 328.125 | -618.0 | -642.2 | 0.7 | 0.208 |
|  |  | ~ dist | 8 | 328.958 | -614.2 | -641.9 | 1.0 | 0.176 |
|  |  |  |  |  |  |  |  |  |
|  | tra.2 | ~ 1 | 7 | 355.571 | -672.9 | -697.1 | 0.0 | 0.502 |
|  |  | ~ dist | 8 | 356.090 | -668.5 | -696.2 | 1.0 | 0.311 |
|  |  |  |  |  |  |  |  |  |
| *V. carlesii* | surv | ~ cage | 9 | -291.077 | 638.1 | 600.2 | 0.0 | 0.615 |
|  |  | ~ cage + dist | 10 | -290.861 | 643.9 | 601.7 | 1.6 | 0.281 |
|  |  |  |  |  |  |  |  |  |
|  | siz.1 | ~ 1 | 7 | 247.057 | -456.2 | -480.1 | 0.0 | 0.335 |
|  |  | ~ dist | 8 | 247.974 | -452.5 | -479.9 | 0.2 | 0.309 |
|  |  | ~ cage | 9 | 248.434 | -448.0 | -478.9 | 1.2 | 0.180 |
|  |  | ~ cage + dist | 10 | 249.270 | -444.2 | -478.5 | 1.6 | 0.153 |
|  |  |  |  |  |  |  |  |  |
|  | siz.2 | ~ dist | 8 | 407.912 | -772.4 | -799.8 | 0.0 | 0.735 |
|  |  |  |  |  |  |  |  |  |
|  | tra.1 | ~ dist | 8 | 253.355 | -463.5 | -490.7 | 0.0 | 0.549 |
|  |  | ~ 1 | 7 | 251.689 | -465.6 | -489.4 | 1.3 | 0.282 |
|  |  |  |  |  |  |  |  |  |
|  | tra.2 | ~ cage | 9 | 333.804 | -619.0 | -649.6 | 0.0 | 0.326 |
|  |  | ~ cage + dist | 10 | 334.790 | -615.6 | -649.6 | 0.0 | 0.321 |
|  |  | ~ cage * dist | 12 | 336.264 | -607.7 | -648.5 | 1.1 | 0.190 |
|  |  |  |  |  |  |  |  |  |
| *R. simsii* | surv | ~ 1 | 7 | -200.137 | 441.3 | 414.3 | 0.0 | 0.503 |
|  |  | ~ dist | 8 | -199.894 | 446.7 | 415.8 | 1.5 | 0.236 |
|  |  |  |  |  |  |  |  |  |
|  | siz.1 | ~ cage | 9 | 246.578 | -445.9 | -475.2 | 0.0 | 0.452 |
|  |  | ~ 1 | 7 | 243.879 | -451.0 | -473.8 | 1.4 | 0.225 |
|  |  | ~ cage + dist | 10 | 246.613 | -440.7 | -473.2 | 1.9 | 0.172 |
|  |  |  |  |  |  |  |  |  |
|  | siz.2 | ~ cage | 9 | 308.938 | -570.6 | -599.9 | 0.0 | 0.361 |
|  |  | ~ 1 | 7 | 306.843 | -576.9 | -599.7 | 0.2 | 0.329 |
|  |  | ~ cage + dist | 12 | 310.968 | -558.9 | -597.9 | 2.0 | 0.134 |
|  |  |  |  |  |  |  |  |  |
|  | tra.1 | ~ 1 | 7 | 226.298 | -417.1 | -438.6 | 0.0 | 0.352 |
|  |  | ~ cage | 9 | 228.203 | -410.7 | -438.4 | 0.2 | 0.320 |
|  |  | ~ dist | 8 | 226.427 | -412.3 | -436.9 | 1.7 | 0.147 |
|  |  | ~ cage + dist | 10 | 228.383 | -406.0 | -436.8 | 1.8 | 0.141 |
|  |  |  |  |  |  |  |  |  |
|  | tra.2 | ~ cage | 9 | 207.447 | -369.2 | -396.9 | 0.0 | 0.468 |
|  |  | ~ cage + dist | 10 | 207.775 | -364.8 | -395.5 | 1.3 | 0.239 |
|  |  |  |  |  |  |  |  |  |
| *Q. serrata* | surv | ~ cage * dist | 13 | -46.672 | 163.7 | 119.3 | 0.0 | 0.817 |
|  |  |  |  |  |  |  |  |  |
|  | siz.1 | ~ 1 | 7 | 6.176 | 7.5 | 1.6 | 0.0 | 0.606 |
|  |  | ~ dist | 8 | 6.351 | 10.0 | 3.3 | 1.7 | 0.265 |
|  |  |  |  |  |  |  |  |  |
|  | siz.2 | ~ cage | 8 | 21.201 | -19.7 | -26.4 | 0.0 | 0.374 |
|  |  | ~ 1 | 6 | 19.045 | -21.1 | -26.1 | 0.3 | 0.320 |
|  |  | ~ cage + dist | 9 | 21.513 | -17.5 | -25.0 | 1.4 | 0.188 |
|  |  |  |  |  |  |  |  |  |
|  | tra.1 | ~ cage | 7 | 10.569 | -1.3 | -7.1 | 0.0 | 0.482 |
|  |  | ~ 1 | 5 | 7.715 | -1.3 | -5.4 | 1.7 | 0.205 |
|  |  |  |  |  |  |  |  |  |
|  | tra.2 | ~ 1 | 5 | 12.661 | -11.2 | -15.3 | 0.0 | 0.379 |
|  |  | ~ cage | 7 | 14.402 | -9.0 | -14.8 | 0.5 | 0.292 |
|  |  | ~ dist | 6 | 13.021 | -9.0 | -14.0 | 1.3 | 0.200 |

**Table S3** Averaged coefficient estimates for subset models of seedling size and trait shifts of *V. carlesii* (Table S2) that were well supported by the data (i.e., ∆AIC was < 2 units greater than the top-ranked model). Models were fitted with restricted maximum likelihood (REML) to obtain unbiased coefficient estimates. Values are means with standard error (SE) in brackets, significant parameter estimates (*P* < 0.05) are in boldface type, marginal significant parameter estimates (*P* < 0.1) are in boldface italic type. Here, ‘half’ and ‘full’ represents half-caged and full-caged treatment, ‘dist’ represents log_2_ distance to edge.

|  | seedling size | | seedling trait | | |  |
| --- | --- | --- | --- | --- | --- | --- |
|  | axis.1 | axis.2 | | axis.1 | axis.2 | |
| intercept | **0.154 (0.024)** | 0.006 (0.007) | | **0.073 (0.028)** | 0.021 (0.016) | |
| half | 0.001 (0.008) |  | |  | 0.021 (0.014) | |
| full | 0.006 (0.011) |  | |  | ***0.030 (0.017)*** | |
| dist | -0.003 (0.004) | **0.004 (0.001)** | | 0.005 (0.005**)** | -0.001 (0.003) | |
| half × dist |  |  | |  | -0.001 (0.002) | |
| full × dist |  |  | |  | -0.002 (0.003) | |
| covariate, early-treat | **1.374 (0.106)** | **0.237 (0.089)** | | **0.530 (0.071)** | **0.237 (0.068)** | |

**Table S4** Averaged coefficient estimates for subset models of seedling size and trait shifts of *R. simsii* (Table S2) that were well supported by the data (i.e., ∆AIC was < 2 units greater than the top-ranked model). Models were fitted with restricted maximum likelihood (REML) to obtain unbiased coefficient estimates. Values are means with standard error (SE) in brackets, significant parameter estimates (*P* < 0.05) are in boldface type, marginal significant parameter estimates (*P* < 0.1) are in boldface italic type. Here, ‘half’ and ‘full’ represents half-caged and full-caged treatment.

|  | seedling size | | seedling trait | |
| --- | --- | --- | --- | --- |
|  | axis.1 | axis.2 | axis.1 | axis.2 |
| intercept | **-0.043 (0.021)** | **0.033 (0.008)** | **0.114 (0.015)** | ***-0.046 (0.024)*** |
| half | <0.001 (0.013) | 0.004 (0.008) | 0.006 (0.012) | **0.036 (0.014)** |
| full | -0.023 (0.019) | -0.008 (0.010) | -0.008 (0.014) | 0.017 (0.014) |
| dist | <0.001 (0.002) | <0.001 (0.001) | 0.001 (0.002) | -0.001 (0.004) |
| covariate, early-treat | **0.919 (0.056)** | **0.349 (0.069)** | **0.310 (0.077)** | **0.483 (0.114)** |

**Table S5** Averaged coefficient estimates for subset models of seedling size and trait shifts of *S. superba* (Table S2) that were well supported by the data (i.e., ∆AIC was < 2 units greater than the top-ranked model). Models were fitted with restricted maximum likelihood (REML) to obtain unbiased coefficient estimates. Values are means with standard error (SE) in brackets, significant parameter estimates (*P* < 0.05) are in boldface type, marginal significant parameter estimates (*P* < 0.1) are in boldface italic type.

|  | seedling size | | seedling trait | |
| --- | --- | --- | --- | --- |
|  | axis.1 | axis.2 | axis.1 | axis.2 |
| intercept | **0.049 (0.010)** | ***0.010 (0.005)*** | **0.084 (0.018)** | -0.010 (0.020) |
| half |  |  | 0.008 (0.010) |  |
| full |  |  | 0.014 (0.014) |  |
| dist |  | 0.001 (0.001) | 0.002 (0.003) | -0.001 (0.003) |
| covariate, early-treat | **0.622 (0.060)** | **0.549 (0.038)** | **0.126 (0.056)** | 0.106 (0.067) |

**Table S6** Averaged coefficient estimates for subset models of seedling size and trait shifts of *Q. serrata* (Table S2) that were well supported by the data (i.e., ∆AIC was < 2 units greater than the top-ranked model). Models were fitted with restricted maximum likelihood (REML) to obtain unbiased coefficient estimates. Values are means with standard error (SE) in brackets, marginal significant parameter estimates (*P* < 0.1) are in boldface italic type.

|  | seedling size | | seedling trait | |
| --- | --- | --- | --- | --- |
|  | axis.1 | axis.2 | axis.1 | axis.2 |
| intercept | 0.044 (0.053) | 0.056 (0.044) | -0.022 (0.062) | 0.043 (0.080) |
| half |  | -0.062 (0.078) | 0.198 (0.159) | 0.056 (0.106) |
| full |  | 0.019 (0.040) | 0.004 (0.071) | -0.015 (0.060) |
| dist | -0.003 (0.010) | 0.002 (0.005) | <0.001 (0.007) | 0.004 (0.012) |
| covariate, early-treat | 0.566 (0.268) | 0.289 (0.318) |  |  |

**Table S7** Results of Pearson’s correlation test. Here, ‘survival’ represents logit-transformed proportion of seedling survival for each species in each cage treatment at each sampling band on each island; ‘siz1.d’ and ‘siz2.d’ represent differences in seedling size between the post- and earlly-treatment stage loadings on Axis 1 and Axis 2 of the seedling size PCoA (Figure 2); ‘tra1.d’ and ‘tra2.d’ represent differences in seedling trait between the post- and earlly-treatment stage loadings on Axis 1 and Axis 2 of the seedling trait PCoA (Figure 3). Significant parameter estimates (P < 0.05) are in boldface type. Note that *Q. serrata* was not included because we did not have seedling trait data at the early-treatment stage of this species.

|  |  | survival | siz1.d | siz2.d |
| --- | --- | --- | --- | --- |
| *S. superba* | tra1.d | -0.044 | 0.023 | **0.156** |
|  | tra2.d | **-0.312** | 0.016 | **-0.155** |
|  |  |  |  |  |
| *V. carlesii* | tra1.d | **-0.134** | **-0.380** | **0.220** |
|  | tra2.d | 0.038 | 0.012 | **-0.283** |
|  |  |  |  |  |
| *R. simsii* | tra1.d | 0.046 | 0.104 | 0.030 |
|  | tra2.d | **0.342** | 0.027 | -0.002 |

**Table S8** Averaged coefficient estimates for subset models of species survival that were well supported by the data (i.e., ∆AIC was < 2 units greater than the top-ranked model; Table S2). Models were fitted with restricted maximum likelihood (REML) to obtain unbiased coefficient estimates. Values are means with standard error (SE) in brackets, significant parameter estimates (*P* < 0.05) are in boldface type, marginal significant parameter estimates (*P* < 0.1) are in boldface italic type. Here, ‘half’ and ‘full’ represents half-caged and full-caged treatment, ‘dist’ represents log_2_ distance to edge, ‘×’ represents interaction between the two factors.

|  | *S. superba* | *V. carlesii* | *R. simsii* | *Q. serrata* |
| --- | --- | --- | --- | --- |
| intercept | 0.77 (0.55) | -0.47 (0.42) | 0.27 (0.40) | 1.50 (7570.21) |
| half | -0.31 (0.47) | -0.35 (0.27) |  | -29.35 (27697.92) |
| full | -0.35 (0.57) | **0.60 (0.26)** |  | -3.82 (7570.22) |
| dist | ***-0.19 (0.11)*** | 0.02 (0.06) | -0.02 (0.08) | -3.93 (7570.22) |
| half × dist | -0.11 (0.14) |  |  | 7.40 (8477.09) |
| full × dist | -0.22 (0.15) |  |  | 3.86 (7579.22) |
| covariate, size.1 | **2.60 (1.28)** | **5.61 (1.40)** | -0.13 (1.51) | -5.10 (3.20) |
| covariate, size.2 | **6.05 (1.64)** | -3.55 (2.38) | **9.64 (2.36)** | 3.84 (5.66) |

**References**

Lichtenthaler, H. K., & Buschmann, C. (2001). Chlorophylls and Carotenoids : Measurement and Characterization by UV-VIS. *Current Protocols in Food Analytical Chemistry*, 1–8.

Struve, D. K. (2009). Tree establishment: A review of some of the factors affecting transplant survival and establishment. *Arboriculture and Urban Forestry*, *35*(1), 10–13.
